# Dysregulation of TGFβ1 Activity in Cancer and Its Influence on the Quality of Anti-Tumor Immunity

**DOI:** 10.3390/jcm5090076

**Published:** 2016-08-31

**Authors:** Kristian M. Hargadon

**Affiliations:** Department of Biology, Hampden-Sydney College, Hampden-Sydney, VA 23943, USA; khargadon@hsc.edu; Tel.: +1-1-434-223-6261

**Keywords:** cancer, TGFβ1, dendritic cell, T cell, immune suppression, immunotherapy

## Abstract

TGFβ1 is a pleiotropic cytokine that exhibits a variety of physiologic and immune regulatory functions. Although its influence on multiple cell types is critical for the regulation of numerous biologic processes in the host, dysregulation of both TGFβ1 expression and activity is frequently observed in cancer and contributes to various aspects of cancer progression. This review focuses on TGFβ1’s contribution to tumor immune suppression and escape, with emphasis on the influence of this regulatory cytokine on the differentiation and function of dendritic cells and T cells. Clinical trials targeting TGFβ1 in cancer patients are also reviewed, and strategies for future therapeutic interventions that build on our current understanding of immune regulation by TGFβ1 are discussed.

## 1. Introduction

The transforming growth factor β (TGFβ) superfamily consists of more than 60 secreted proteins that play critical roles in regulating diverse biological processes during embryonic development and in adults. In particular, members of the TGFβ subfamily, of which TGFβ1 is the most well-studied isoform in mammals, have been shown to regulate various aspects of cell proliferation, differentiation, adhesion, migration, angiogenesis, apoptosis, survival and immune surveillance [1]. Because strict regulation of these processes is vital to maintaining cellular homeostasis and tissue integrity, dysregulation of TGFβ1 expression and activity has significant pathologic consequences and contributes to a number of disease states, including many cancers [2]. This review focuses on the role of TGFβ1 dysregulation in cancer-associated immune suppression and highlights how our current understanding of TGFβ1-mediated tumor immune escape is driving therapeutic interventions to target this pathway in the treatment of cancer. 

## 2. TGFβ1 Expression and Signaling

TGFβ1 expression has been observed in a variety of cell types and may be driven by various stimuli that include growth factors, hormones, cytokines, interaction with apoptotic cells and TGFβ1 itself [3,4,5,6,7,8]. Transcriptional activation of the *Tgfb1* gene is mediated by signaling through the RAS/MAPK, PI3K or PKC signaling pathways [9,10,11], and the androgen receptor, as well as AP-1, NFκb, Sp1 and STAT3 transcription factors have all been shown to bind to promoter elements within this gene and induce its expression [3,4,12,13,14,15,16]. TGFβ1 is initially translated as a latent precursor protein that must undergo extensive processing before becoming active. Details of this processing have recently been reviewed in depth elsewhere [17]. In short, proteolytic cleavage of homodimerized pro-TGFβ1 results in mature TGFβ1 peptide that is coupled to latency associated peptide (LAP). This small latent complex associates with latent TGFβ binding protein (LTBP) to form a large latent complex that is secreted into the extracellular space. Bioactive TGFβ1 protein is produced when the large latent complex and LTBP are cleaved from the mature protein in acidic environments or by a variety of mediators that include thrombospondin-1, integrins, reactive oxygen species and various proteases.

**Figure 1:**
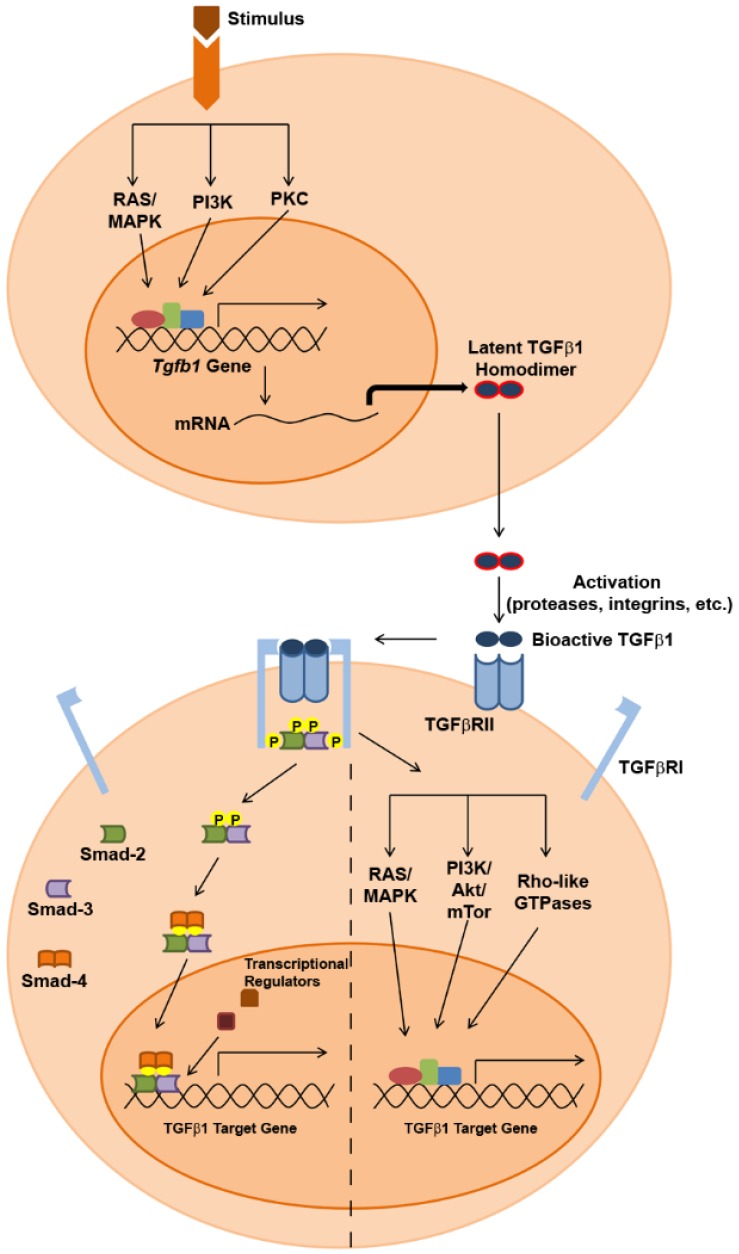
Summary of TGFβ1 expression and SMAD-dependent/SMAD-independent signaling pathways.

Following its activation, TGFβ1 initiates signaling in a target cell (Figure 1) by binding as a homodimer to type II TGFβ receptors (TGFβRII), constitutively-active serine/threonine kinase receptors that undergo a conformational change upon ligand binding that enables recruitment of type I TGFβ receptors (TGFβRI). Phosphorylation of TGFβRI by TGFβRII within a heterotetrameric complex then activates downstream signaling events, which can involve both SMAD-dependent and SMAD independent pathways. In SMAD-dependent signaling, phosphorylated TGFβRI recruits and phosphorylates the receptor-activated SMADs (R-SMADs), SMAD2 and SMAD3, which in turn interact with a co-SMAD known as SMAD4. This heterotrimeric complex then translocates to the nucleus, where it interacts with various co-activator/co-repressor proteins to regulate the expression of target genes, and cell type-dependent responses to TGFβ1 signaling are influenced by the particular SMAD cofactors that associate with the R-SMAD/co-SMAD complex in specific cell types [18]. Additionally, SMAD-dependent TGFβ1 signaling can also result in epigenetic regulation of gene expression. One recent study suggests that SMAD2 is able to silence gene expression through recruitment of DNA methyltransferases to promoter regions of target genes [19], and others have shown that TGFβ1-SMAD signaling can promote histone acetylation and chromatin remodeling [20,21]. Finally, SMAD-independent signaling through the TGFβRI/II receptor system can be achieved through activation of the RAS/MAPK and PI3K/Akt/mTOR pathways, as well as through activation of the Rho-like family of small GTPases. Figure 1 provides a summary of these TGFβ1 signaling pathways, which have been described more thoroughly in several recent reviews [17,18,22,23].

## 3. Overexpression of TGFβ1 in Cancer

**Figure 2:**
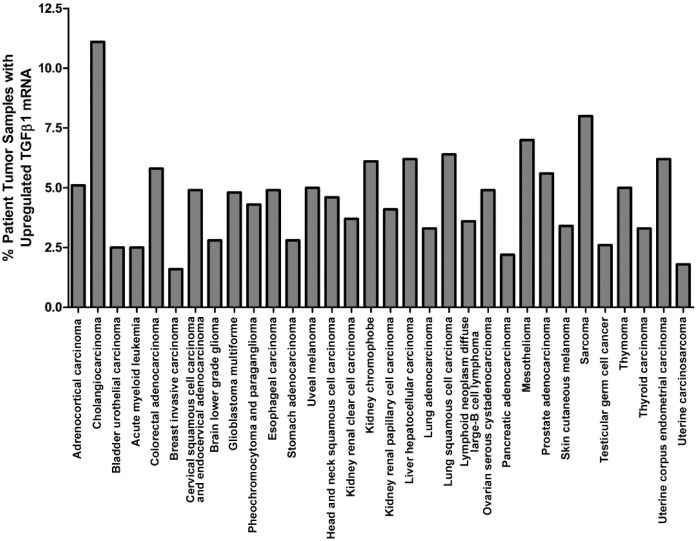
TGFβ1 mRNA upregulation in tumor samples as determined by RNA Seq V2 RSEM (z-score threshold = 2.0). Results were obtained from an interrogation of TCGA, provisional data on 5 June 2016 and are based on data generated by the TCGA Research Network: http://cancergenome.nih.gov/.

Originally recognized for its potent inhibition of cell growth [24,25], TGFβ1 has been shown to mediate anti-proliferative effects on many cell types by suppressing c-Myc expression [26,27] and altering the expression and activity of cyclin-dependent kinases (CDK) and CDK inhibitors that regulate progression through the cell cycle [28,29,30,31,32]. Paradoxically, despite its ability to inhibit cell proliferation, TGFβ1 is highly expressed within the tumor tissue of many cancer patients, as is evidenced by a recent interrogation of The Cancer Genome Atlas using the cBioPortal for Cancer Genomics [33,34], which revealed upregulation of TGFβ1 mRNA levels in tumors from various cancer types (Figure 2). Several other independent studies have also reported elevated TGFβ1 expression within tumor tissue or plasma of patients with various cancers. Many of these studies have correlated increased TGFβ1 expression levels with advanced tumor stage and diminished patient survival, and elevated expression of TGFβ1 in these patients is associated with several specific aspects of tumor progression that include epithelial-mesenchymal transition (EMT), angiogenesis, tissue invasion and metastasis [35,36,37,38,39,40,41,42,43]. Importantly, increased TGFβ1 levels in cancer may arise not only from enhanced expression of this cytokine by tumor cells themselves, but also by recruitment into the tumor microenvironment of TGFβ1-producing cancer-associated cells that include stromal fibroblasts, tumor-associated macrophages, dendritic cells, and immature myeloid cells [44,45,46,47]. The dichotomy of TGFβ1’s anti-proliferative yet pro-tumor activities can be explained by the acquisition of tumor cell resistance to the negative regulatory effects of this cytokine during tumor progression. Indeed, decreased expression of TGFβRI/II receptors or mutations in these proteins that abrogate TGFβ1 signaling in tumor cells have been observed in many cancer types [48,49,50,51,52,53]. Similarly, tumor cells may escape growth inhibition by autocrine/paracrine TGFβ1 signaling through alterations to SMAD signaling components. Mutations and deletions of genes encoding SMAD proteins have been observed in some cancer cells [54,55,56,57], while others have been shown to exhibit post-translational modifications to SMAD proteins that promote their cytoplasmic retention and degradation [58,59]. Still other tumor cells have been reported to overexpress the SMAD7 inhibitory SMAD (I-SMAD) that competitively inhibits TGFβ1-mediated SMAD signaling [60,61]. Any of these alterations to TGFβ1 signaling pathway components can shield tumor cells from the growth inhibitory effects of TGFβ1 while still allowing the tumor-promoting activities of this cytokine to be triggered in other cells within the milieu of the tumor microenvironment. Moreover, tumor cells that ultimately escape SMAD-dependent growth inhibitory signals from TGFβ1 do not necessarily become totally unresponsive to this cytokine; rather, many tumors evolve to shift TGFβ1 signals along pro-oncogenic pathways. In this light, a recent microarray analysis of gene expression in a TGFβ1-treated lung epithelial cell line versus a TGFβ1-treated lung adenocarcinoma cell line revealed differential regulation of gene expression by this cytokine in normal versus tumor cells, and the unique induction of several specific genes with tumor-promoting function by TGFβ1 in tumor cells was impaired by multiple MAPK pathway component inhibitors [62]. Similarly, other studies have shown that retention of SMAD-independent TGFβ1 signaling in late-stage tumors contributes to their progression by promoting EMT, loss of cell adhesion and increased migration/invasion [63]. Therefore, the combination of altered TGFβ1 signaling within tumor cells and traditional TGFβ1 signaling within other cell types in the tumor microenvironment results in pleiotropic effects by this cytokine that create a “perfect storm” ideally suited for tumor progression. In addition to its promotion of several of the cancer hallmarks described above (which have been reviewed extensively in many of the articles previously cited), TGFβ1 has also been shown to compromise the function of several cells involved in anti-tumor immune responses and, therefore, contributes significantly to tumor immune escape.

## 4. TGFβ1 in Tumor Immune Suppression and Escape

### 4.1. TGFβ1 Influence on the Function of Dendritic Cells and Their Hematopoietic Precursors 

Dendritic cells (DC) are a population of innate immune cells derived from both lymphoid and myeloid progenitors that play key roles in regulating the activity of other immune cells, particularly T lymphocytes. Following their differentiation from hematopoietic precursors in the bone marrow, DC take up residence in both lymphoid and peripheral tissues, where they are involved in immune surveillance. As immature cells in the steady state, DC are highly phagocytic and sample antigen from various sources, though presentation of antigen by immature DC to T lymphocytes results in either immunologic ignorance or tolerance to such antigen [64,65,66]. On the other hand, following the encounter with various mediators that may include pathogen- or danger-associated molecular patterns, inflammatory stimuli and CD40L, immature DC become mature, activated cells that acquire potent immune stimulatory functions, which arise from their upregulation of antigen:MHC complexes, costimulatory molecules, “signal 3” cytokines and chemokines, all of which are involved in the activation and recruitment of T cells and other immune effectors into an immune response [67,68]. 

**Figure 3:**
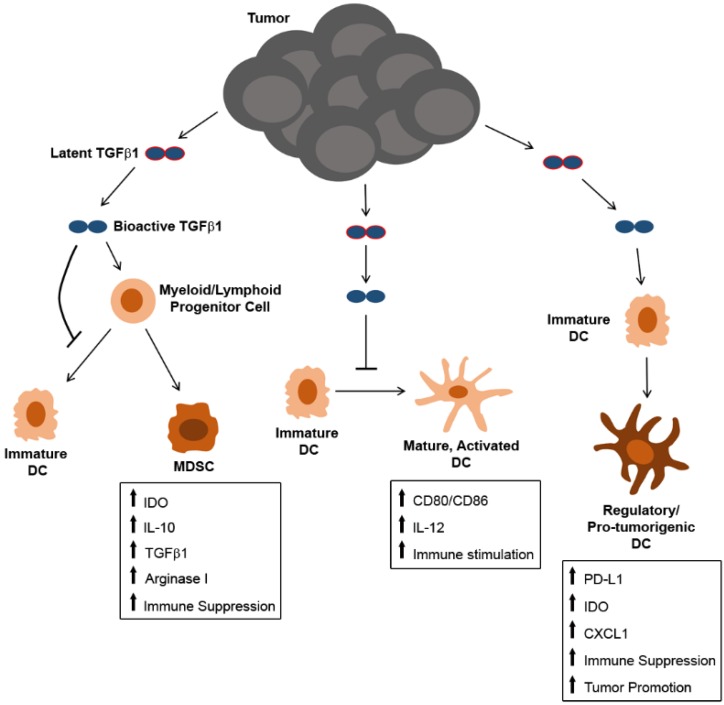
Overview of mechanisms by which tumor-derived TGFβ1 may influence the differentiation and function of DC and their precursors.

TGFβ1 is known to influence DC differentiation and function in a number of ways (Figure 3). In vitro analyses of both bone marrow- and monocyte-derived DC have shown that TGFβ1 can inhibit the development of DC from hematopoietic precursors, and those DC that do develop in the presence of TGFβ1 retain an immature phenotype characterized by low MHC class II and costimulatory molecule expression and poor T cell stimulatory activity [69,70,71]. Others have shown that TGFβ1 alters the differentiation program of DC precursors, leading to the development of myeloid-derived suppressor cells (MDSC) [72,73] that are known to promote tumor outgrowth through a variety of mechanisms [74,75]. In addition to its impact on the differentiation of hematopoietic precursors into DC, TGFβ1 has also been shown to interfere with the maturation and activation of fully-differentiated DC, as well. It has been shown to block the expression of the costimulatory molecules CD80 and CD86, as well as the “signal 3” cytokine IL-12 in in vitro-generated Langerhans DC [76]. Furthermore, studies using transgenic mouse models have shown the tolerogenic effects of TGFβ1 on DC in vivo, as well. For example, mice expressing a DC-restricted, CD11c promoter-driven dominant negative TGFβRII receptor that lacks the kinase domain necessary for signal transduction produce DC that are resistant to TGFβ1 tolerization, resulting in aberrant, DC-dependent autoimmune T cell activation [77]. Similar findings have been reported in double transgenic mice expressing Cre recombinase under control of the CD11c promoter and a loxP-flanked *Tgfbr2* gene. Inducible knockout of TGFβRII specifically in DC of these mice leads to severe autoimmunity that is partially attributed to the inability of DC to support regulatory T cell (Treg) differentiation and expansion [78]. With specific regard to tumor-derived TGFβ1, recent work from our laboratory has shown that TGFβ1 in melanoma tumor-conditioned media also alters the maturation and activation of fully-differentiated tissue-resident DC. Although these tumor-altered DC could still activate CD8+ T cells in an ex vivo setting, they exhibited modified cytokine and chemokine expression profiles that correspond to a pro-tumorigenic phenotype. This phenotype could be partially reversed by *Tgfb1* gene silencing in melanoma cells prior to ex vivo culture of tissue-derived DC in tumor-conditioned media. Of particular note among the alterations to DC function observed in our model, melanoma-derived TGFβ1 promoted DC secretion of CXCL1, a known macrophage chemoattractant, and enhanced the expression of this chemokine by lung-resident DC in mice bearing lung metastatic melanoma lesions correlated with an increase in M2-like macrophages at this site [79]. Recent findings from several other groups have also shown that TGFβ1 induces tumor-promoting functions in DC. For instance, Belladonna et al. demonstrated that TGFβ1 promotes indoleamine 2,3-dioxygenase (IDO) expression and tolerogenic activity in both CD8− and CD8+ murine DC subsets [80], and IDO-producing regulatory DC play critical roles in anti-tumor immune suppression in various cancer types [81]. Alternatively, studies in a murine ovarian cancer model have shown that tumor-derived TGFβ1 can induce PD-L1 expression on DC that suppress T cell proliferation [82], and in patients with highly aggressive triple negative breast cancer, TGFβ1 has been shown to induce regulatory plasmacytoid DC that exhibit diminished type I IFN production and that promote expansion of CD4+ Tregs [83,84]. Collectively, these deleterious effects of TGFβ1 on DC development and function significantly compromise the quality of anti-tumor immune responses and can be a major contributing factor to tumor immune escape. 

### 4.2. TGFβ1 Influence on Tumor-Associated Macrophages and Neutrophils 

In addition to DC, other immune cell populations of myeloid origin are also known to be influenced by TGFβ1. In particular, macrophages exposed to TGFβ1 have been shown to acquire an M2-like phenotype characterized by a number of tumor-promoting functions, including the ability to promote angiogenic activity, suppress T cell proliferation and induce CD4+ FOXP3+ Treg differentiation [85,86,87,88]. Importantly, several clinical studies have reported that patient tumors are often infiltrated by a large number of macrophages, particularly those exhibiting an anti-inflammatory, immune suppressive M2-like phenotype, and such accumulation is a negative prognostic indicator in cancer patients [89,90,91,92,93]. Similarly, TGFβ1 has been suggested to polarize tumor-associated neutrophils (TAN) from an N1- to an N2-like phenotype, as the blockade of TGFβ1 in several murine tumor models enhances cytotoxic activity and proinflammatory cytokine production by tumor-infiltrating neutrophils, whereas the depletion of neutrophils in the context of TGFβ1-expressing tumors diminishes tumor outgrowth and is associated with enhanced intratumoral CD8+ T cell activation [94]. As seen with M2-like macrophage accumulation within tumors, high levels of tumor-associated neutrophils in cancer patients are also associated with disease progression and poor survival [95,96]. Moreover, not only are these pro-tumor immune populations induced by TGFβ1, but at least in the case of tumor-associated macrophages (TAM), these cells can become potent producers of TGFβ1 themselves [97,98], thus further contributing to the immunosuppressive and tumor-promoting effects of this cytokine within the tumor microenvironment during cancer progression. 

### 4.3. TGFβ1 Influence on T Cells 

Because of their ability to recognize highly specific antigens on the surface of a target cell, T lymphocytes have the potential to serve as potent immunologic effectors against tumor cells. Indeed, studies reporting increased tumor incidence in RAG^−/−^ mice and mice deficient in the cytolytic mediator perforin highlight the role of T lymphocytes in immune surveillance against tumors [99,100,101]. Similar reports of increased tumor incidence in immunocompromised patients and transplant patients receiving immunosuppressive drug therapy [102,103], in conjunction with observations of spontaneous tumor regression in patients exhibiting natural or therapy-induced anti-tumor T cell responses [104,105], have offered support for the critical role of T cells in tumor eradication in humans, as well. However, despite the ability of T cells to eradicate tumors in some cases, many cases of tumor progression are associated with the induction of tumor-specific T cell dysfunction [106,107,108,109,110], thus highlighting the significance of T cell suppression as a contributing factor to tumor immune escape. 

TGFβ1 is a well-characterized regulator of T cell differentiation and function. Transgenic mouse models that employ cell type-specific promoters to restrict the expression of a dominant negative TGFβRII receptor to CD4+ or CD8+ T cells have enabled in vivo analyses of the effects of TGFβ1 on T cells and have revealed that this cytokine directly inhibits T cell proliferation and activation of Th1/cytotoxic differentiation programs, while at the same time promoting the survival of Tregs [111,112]. In the context of the tumor microenvironment, therefore, various sources of TGFβ1 may contribute to T cell dysfunction and ultimately limit the efficacy of anti-tumor immune responses mediated by these cells. To this point, in a murine model of prostate cancer, conditional knockout of TGFβRII in adoptively-transferred tumor-specific CD8+ T cells resulted in reduced apoptosis, increased proliferation and effector activity and delayed induction of dysfunction in these cells as compared to adoptively-transferred cells in which TGFβ1 signaling was not abrogated [113]. Although the source of TGFβ1 was not investigated in this study, others have shown that both tumors and tumor-associated cells contribute to TGFβ1-mediated anti-tumor T cell dysfunction. For instance, in the EG7 murine thymoma tumor model, membrane-bound TGFβ1 expressed on tumor apoptotic bodies has been shown to inhibit anti-tumor cytotoxic T lymphocyte (CTL) responses through the induction of CD8+ T cell anergy, while at the same time promoting the development of IL-10-producing CD4+ Tregs that inhibit CD8+ T cell proliferation and differentiation into CTL [114]. Anti-tumor T cell responses have also been shown to be inhibited by TGFβ1 derived from cells of myeloid origin [45,115]. With regard to CD4+ Tregs, TGFβ1 is known to promote the differentiation of these cells through induction of FOXP3 expression [116], and various tumor-associated cell types that include mesenchymal stem cells, myeloid-derived suppressor cells (MDSC), and DC have all been shown to produce TGFβ1 and induce either the proliferation or differentiation of Tregs [46,117,118,119]. Finally, not only does TGFβ1 contribute to the development of Tregs, but Tregs themselves suppress T cell function through TGFβ1 [120], and blockade of TGFβ1 signaling in CD8+ T cells has been shown to prevent Treg-mediated suppression of anti-tumor immunity in a murine colon carcinoma model [121]. 

**Figure 4:**
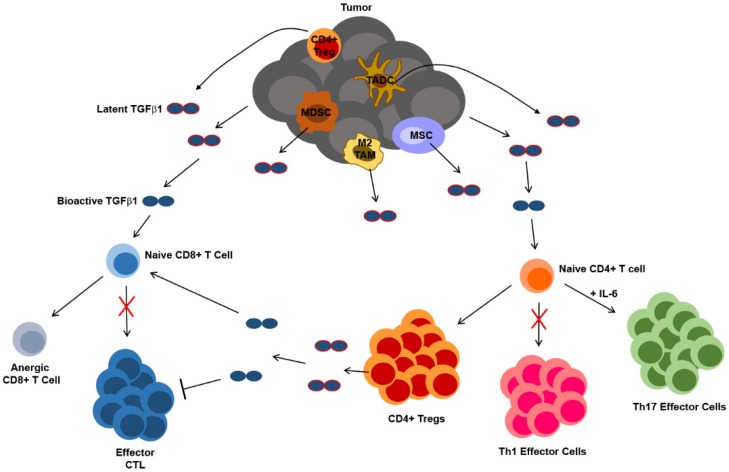
Summary of the mechanisms by which TGFβ1 derived from tumors and tumor-associated cells may influence the differentiation and function of CD4+ and CD8+ T lymphocytes. TADC = tumor-associated dendritic cell, TAM = tumor-associated macrophage, MSC = mesenchymal stem cell, MDSC = myeloid-derived suppressor cells, CTL = cytotoxic T lymphocyte, Treg = regulatory T cell.

In addition to inducing the differentiation of CD4+ T cells into immunosuppressive Tregs, TGFβ1 can also act in concert with IL-6 to promote CD4+ T cell differentiation along a Th17 pathway [122]. Although many studies have demonstrated anti-tumor functions of Th17 cells (reviewed in [123]), others have shown that Th17 cells exhibit pro-tumor functions in certain contexts. In both the B16 melanoma and MB49 bladder carcinoma murine tumor models, IL-17 produced by CD4+ T cells activated STAT3 in both tumor and stromal cells, leading to the expression of both anti-apoptotic and pro-angiogenic proteins within the tumor microenvironment [124]. In the EG7 murine thymoma model, IL-17 is required for the development and pro-tumor functions of MDSC [125]. IL-17 also enhances the proliferation of human colorectal cancer cell lines in vitro, and Th17 cells are enriched in tumor-infiltrating leukocyte (TIL) populations of colorectal cancer patients [126]. Likewise, in another study with multiple myeloma patients, the number of Th17 cells was increased in both the blood and bone marrow, and IL-17 was shown to stimulate the growth of human multiple myeloma cell lines in vitro and in a murine xenograft model in vivo [127]. Other clinical studies have revealed that Th17 cell infiltration and IL-17 expression levels in tumors are associated with tumor progression and poor survival in patients with gastric cancer and hepatocellular carcinoma [128,129]. Therefore, the influence of TGFβ1 on helper and cytotoxic T cell differentiation and function, which is summarized in Figure 4, can not only impede the anti-tumor effector functions mediated by these cells, but it can also confer tumor-promoting activity in some T cell populations that further drives tumor growth and metastasis. 

### 4.4. TGFβ1 Influence on Natural Killer Cells

Like effector CTL, natural killer (NK) cells may also serve as potent mediators of anti-tumor immunity. Instead of responding to specific tumor antigens as CTL do, NK cells instead respond to targets that have either downregulated MHC class I molecules or upregulated stress-associated markers, characteristics often exhibited by cancer cells during tumor progression [130]. However, in addition to its suppressive effects on the cytotoxic activity of CD8+ T cells, TGFβ1 has similarly been shown to inhibit cytotoxic effector functions in NK cells, as well. In this regard, in a murine model of liver cancer TGFβ1 expressed on the membrane of MDSC inhibited expression of the activating receptor NKG2D on hepatic NK cells, suppressed NK cell IFNγ secretion and cytotoxicity and rendered NK cells anergic to activating stimuli [131]. TGFβ1 has also been implicated in STAT3-dependent suppression of NK cell cytotoxic activity in a murine model of hepatocellular carcinoma [132], and it is partially responsible for the downregulation of NKG2D expression and cytolytic activity by NK cells in an orthotopic model of head and neck squamous cell carcinoma [133]. One group has shown that TGFβ1-mediated suppression of NK cell cytolytic activity is attributed to its induction of the microRNA miR-183, which silences the expression of the DAP12 adapter protein required to transmit activating signals for lytic granule mobilization [134]. Most recently, Viel et al. have shown that TGFβ1 signaling in both murine and human NK cells inhibits their activation by repressing the mTOR pathway and that deletion of TGFβRII on NK cells restores mTOR signaling and promotes their ability to limit metastasis in multiple murine tumor models [135]. Importantly, these findings in preclinical settings have been supported by studies involving cancer patients, as well. In both lung and colorectal cancer patients, elevated plasma TGFβ1 levels correlated with decreased NKG2D expression on freshly-isolated NK cells, and downregulation of NKG2D on NK cells that were derived from healthy donors and subsequently cultured with plasma from cancer patients could be prevented by the addition of neutralizing anti-TGFβ1 monoclonal antibodies to the ex vivo cultures [136]. Likewise, TGFβ1 expression levels by tumor cells in patients with advanced gastric adenocarcinoma are inversely correlated with the cytolytic activity of NK cells isolated from the ascites and peripheral blood of these patients [137]. Taken together, these results demonstrate that TGFβ1 is a key immunosuppressive factor that confers tumor cell resistance to NK cells. In conjunction with the aforementioned discussion of TGFβ1’s immunosuppressive effects on CTL, these findings indicate that TGFβ1 is capable of compromising both of the major cytolytic mediators associated with anti-tumor immune responses, and its influence on other cells of the immune system ultimately contributes not only to the dysfunction of these cytolytic effector cell populations, but also to the overall promotion of tumor growth and metastasis. 

## 5. Strategies for Interfering with TGFβ1-Mediated Suppression of Anti-Tumor Immunity

With the emergence of data documenting the impact of TGFβ1 on the activation and function of various immune cell populations in both preclinical models and cancer patients, significant efforts have recently been made on developing therapeutic strategies for interfering with TGFβ1-mediated suppression of anti-tumor immune responses. Several approaches that either block ligand-receptor interactions or inhibit intracellular signaling cascades have been employed in a non-specific manner to systemically block TGFβ1 from influencing the behavior of target cells bearing receptors for this regulatory cytokine. Administration of a TGFβRI kinase inhibitor augmented the immunogenicity and anti-tumor efficacy of adenoviral vector-based vaccines in multiple murine lung tumor models, promoting increased tumor infiltration of macrophages, NK cells and CD8+ T cells [138]. Similar therapeutic benefits were observed in multiple murine mesothelioma tumor models following administration of a soluble TGFβRII chimeric protein designed to neutralize TGFβ1 (and TGFβIII) and thereby abrogate its signaling in target cells. Treatment of mice bearing established mesothelioma tumors with this chimeric “decoy” receptor delayed tumor outgrowth, and this control was associated with improved anti-tumor CD8+ T cell responses; specifically, mice treated with this soluble TGFβRII protein displayed enhanced cytolytic activity in splenic CTL and increased CD8+ T cell infiltration of tumors, whereas no therapeutic benefit was observed in mice depleted of CD8+ T cells prior to treatment [139]. Neutralization of all three TGFβ isoforms via administration of the 1D11.16 monoclonal antibody has also been shown to significantly enhance the efficacy of a prophylactic irradiated tumor vaccine in the CT26 colorectal cancer model, and like the aforementioned mesothelioma studies, the therapeutic benefit of this treatment was dependent on CD8+ T cells, as well [140]. Still another approach involving a fusion protein known as FIST, which consists of the soluble extracellular domain of TGFβRII linked to the immunostimulatory cytokine IL-2, has been shown to inhibit both pancreatic cancer and B16 melanoma outgrowth; while this inhibition is likely at least partially attributable to the anti-angiogenic effects of FIST, it is also associated with enhanced immune cell recruitment to tumor sites; and a soluble factor derived from NK cells was implicated in FIST-associated tumor control [141]. Additionally, as an alternative to these approaches that interfere directly with TGFβ1 or the signaling mediated by this protein, it is also possible to silence expression of the *Tgfb1* gene so that the protein cannot be synthesized at normal levels. Such an approach has been implemented successfully in the B16 melanoma model, as administration of TGFβ1 siRNA in conjunction with a DC vaccine significantly enhanced the control of this tumor and was associated with a decrease in Tregs at the tumor site [142].

Despite the promise of the aforementioned approaches and similar strategies that act to systemically block TGFβ1 signaling, because of the pleiotropic regulatory activities of TGFβ1, there is concern that long-term systemic therapies targeting this pathway might have unintended and deleterious side effects [143,144,145]. Recently, advances in genetic engineering have enabled creative strategies to overcome this limitation and disrupt TGFβ1 signaling in specific cell populations, and several immunotherapeutic maneuvers have been developed with the aim of preventing TGFβ1-mediated suppression of either: (1) endogenous immune cell populations in the host; or (2) exogenous cells delivered as part of anti-cancer immunizations. For instance, TGFβ1 resistance has been introduced specifically into CD8+ T cells ex vivo by infection with a retrovirus encoding a dominant negative TGFβRII, and adoptive transfer of these T cells into tumor-bearing hosts led to significant reduction in primary tumor size and pulmonary metastases in the TRAMP-C2 transgenic adenocarcinoma of the mouse prostate model [146,147,148]. Improved anti-tumor CTL activity has also been observed in adoptively-transferred dominant negative TGFβRII-expressing T cells in a murine medulloblastoma tumor model [149]. Interestingly, tumor antigen-specific CD4+ and CD8+ T cells retrovirally transduced to express the dominant negative TGFβRII each provided enhanced tumor control when transferred into B16 melanoma-bearing mice, but no therapeutic benefit resulted when adoptively-transferred T cells had been transduced with retrovirus encoding soluble “decoy” TGFβRII proteins that could neutralize TGFβ1 signaling not only in T cells, but also in bystander cell populations [150]. These findings underscore the benefits of abrogating TGFβ1 signaling specifically in T lymphocytes as opposed to multiple targets in an undefined way, particularly as non-specific neutralization of TGFβ1 might interfere with its growth inhibitory effects on tumor cells that have not yet evolved to escape anti-proliferative signals conferred by TGFβ1. Moreover, the promise of inducing TGFβ1 resistance specifically in T cells is further highlighted by preclinical studies using a severe combined immunodeficient SCID xenograft model of Epstein-Barr virus (EBV)-positive lymphoma, which have revealed that EBV-specific CTL derived from patients and engineered to express dominant negative TGFβRII also confer enhanced tumor protection as compared to TGFβ1-sensitive CTL [151]. Importantly, concerns about aberrant lymphoproliferation of TGFβ1-resistant CTL have been addressed in a non-tumor murine model using human papillomavirus E7-specific CTL, and spontaneous proliferation of dominant negative TGFβRII-engineered CTL did not occur in the absence of antigenic stimulation [152].

Specific ablation of TGFβ1 signaling has also been achieved in DC that have been utilized for the purpose of cancer vaccination. Introduction of the dominant negative TGFβRII into DC renders these cells resistant to TGFβ1-mediated suppression, and immunization of mice with tumor lysate-pulsed DC engineered in this way led to robust anti-tumor CTL responses that inhibited tumor growth and enhanced the survival of mice bearing TRAMP-C2 prostate tumors [153]. Nearly identical results were reported when dominant negative TGFβRII-expressing DC were used to immunize mice bearing renal carcinoma metastases in the lungs [154]. As an alternative to retroviral transduction as a means of introducing TGFβ1 resistance in DC, siRNA-mediated gene silencing of the TGFβ receptor in exogenous bone marrow-derived DC also significantly improved the immunogenicity of these cells in a murine model of cervical cancer expressing the HPV-16 E7 antigen [155]. Recent work has also shown potential promise for the targeting of *Tgfb1* (and other genes) in endogenous tumor-associated DC. Using nanocomplexes encapsulating miR-155 miRNA, Cubillos-Ruiz et al. demonstrated that preferential engulfment of these complexes by tumor-associated DC in vivo led to a reprogramming of DC function from one of immunosuppression to one of immune stimulation that in turn enhanced anti-tumor T cell effector function and improved the control of established ovarian carcinoma [156]. miR-155 delivery to tumor-associated DC in this model led to several changes in the transcriptome of these cells, including the silencing of *Tgfb1* and other genes involved in the TGFβ1 signaling pathway. While these changes are likely not solely responsible for the reversal of DC function in this setting, these results highlight the potential for specific targeting of DC in situ, and future advances in our understanding of both gene regulation and cell type-specific delivery methods will undoubtedly allow scientists to fine-tune approaches for interfering with TGFβ1 production by, or signaling within, particular cell populations. Moreover, though it has yet to be explored with respect to TGFβ1’s influence on anti-tumor immune responses, advances in genome editing strategies, such as CRISPR-Cas9 approaches that can be tailored to target gene function at the level of DNA, offer exciting promise for the permanent disruption of genes in specific cells and, therefore, might have an advantage over gene silencing approaches that confer only a temporary diminution in target gene expression. Particularly in the context of exogenous DC or CTL used for cancer vaccination and adoptive transfer therapies, it is appealing to speculate that permanent disruption of the *Tgfbr2* gene by genome editing might further improve the immunogenicity of these cells as compared to cells altered by less permanent gene silencing approaches. Such genome editing would also likely carry advantages over viral vector-based methods of introducing into these immune cell populations a dominant negative TGFβ receptor, which can confer permanent resistance to TGFβ1 signaling, but which may allow for random integration of viral vectors into the genome and functional disruption of unintended genes or the expression of viral antigens in transduced cells that ultimately flag them for destruction by the immune system, thus preventing any long-term immunologic benefit.

## 6. Clinical Trials Targeting TGFβ1 in the Context of Cancer Immunotherapy

The accumulation of data over the last 30 years that TGFβ1 plays several key roles in the progression of cancer and increasing evidence from animal and preclinical studies demonstrating the anti-tumor efficacy of many strategies that interfere with TGFβ1 activity have together made TGFβ1 an attractive target for cancer therapy in patients. Indeed, several TGFβ1 pathway inhibitors have been or are currently being tested in clinical trials for various cancer types. These inhibitors include monoclonal antibodies to TGFβ1 or TGFβ receptors that aim to prevent ligand-receptor interactions, TGFβ1 peptide inhibitors and small molecule inhibitors that aim to block TGFβ1 signal transduction at the intracellular level. Clinical trials utilizing these inhibitors have recently been reviewed elsewhere [157]. Only one of these inhibitors (GC1008, a monoclonal pan-TGFβ neutralizing antibody otherwise known as fresolimumab) has been evaluated for its impact on immune cell populations in cancer patients, and it was shown to have no impact on Treg frequency or the expression of activation markers on CD4+ T cells, CD8+ T cells or NK cells in patients with malignant pleural mesothelioma (MPM). Although this study did demonstrate increased levels of serum antibodies that could react with MPM tumor lysates (but that could not bind live MPM cell lines) following treatment, the number of patients ultimately enrolled in the study was limited due to discontinuation of antibody development for oncology indications [158]. While some TGFβ1 inhibitors have shown promise in early-phase trials, others have also been abandoned [157]. As alluded to in the previous section, though, strategies that aim to specifically target TGFβ1 signaling in immune cell populations might be more advantageous than TGFβ pathway inhibitors that block signaling systemically, and several ongoing clinical trials are currently incorporating such strategies into novel cancer immunotherapies (Table 1). 

Adoptive transfer of tumor antigen-specific CTL has become one of the most promising immunotherapies for the treatment of cancer. Because TGFβ1 is known to compromise CTL effector function, several trials have been designed to investigate whether introducing TGFβ1 resistance into adoptively-transferred CTL can boost the anti-tumor efficacy of these cells. Following up on the promise of the preclinical studies described above, trials involving dominant negative TGFβ receptor-expressing CTL (DNR-CTL) are currently underway for several cancers. TGFβ-resistant CTL specific for LMP antigens of EBV are being used for adoptive transfer therapy of patients with EBV+ lymphoma. A similar trial comparing the adoptive transfer of EBV-specific DNR-CTL with or without chemotherapy-induced lymphodepletion is also ongoing for patients with EBV+ nasopharyngeal carcinoma. In an approach to target multiple tumor antigens with the same adoptively-transferred CTL, a chimeric antigen receptor (CAR) specific for the human epidermal growth factor receptor 2 (HER2) has been introduced into EBV-specific DNR-CTL generated from the blood of EBV seropositive patients for investigation in patients with HER2+ malignancies. Human papilloma virus-associated cancers are also being targeted with HPV E7 antigen-specific DNR-CTL. Finally, in contrast to the previously described approaches in which CTL are generated from patient blood, tumor-infiltrating lymphocytes from patients with metastatic melanoma are also being engineered to express the dominant negative TGFβRII prior to adoptive transfer therapy in conjunction with high-dose IL-2.

Clinical trials incorporating strategies to limit TGFβ1-mediated immune suppression have not been restricted solely to adoptive T cell transfer therapies either. The Vigil™ (formerly known as FANG™) vaccine, which consists of autologous tumor cells transfected with a plasmid vector encoding both granulocyte-macrophage colony-stimulating factor (GM-CSF) and a bi-functional shRNA designed to silence expression of the furin convertase that activates both TGFβ1 and TGFβ2, has already been validated in a phase I trial involving patients with various late-stage cancers [159]. In this trial, the Vigil™ (FANG™) vaccine was well tolerated with minimal adverse events, and the expression of TGFβ1 and TGFβ2 was decreased 93.5% and 92.5%, respectively. Survival was significantly enhanced in patients receiving ≥4 vaccines, and 50% of this group’s patients whose PBMC were tested for reactivity against autologous tumor cells showed an increase in IFNγ-producing cells by Enzyme-Linked ImmunoSpot ELISPOT analysis. Similar immunologic and clinical benefits from this vaccine have since been reported in follow-up studies [160,161] and in a phase I trial involving patients with advanced Ewing sarcoma [162,163]. Although the exact mechanism of improved immune reactivity achieved by Vigil™ (FANG™) vaccination is less clear than DNR-CTL adoptive transfer therapies in which TGFβ resistance is introduced specifically into T cells, it is likely that indirect TGFβ knockdown via this approach diminishes the suppression of DC, whose recruitment to and activation at the vaccination site is also enhanced by GM-CSF secreted by the engineered autologous tumor cells. By employing autologous tumor cells that have the potential to promote immune reactivity against several patient-specific tumor antigens, the “triad” functionality of Vigil™ (FANG™) vaccination is achieved, creating possible advantages over adoptive cell transfer therapies that target only a single tumor antigen and that are restricted to only a subset of patients whose tumors test positive for such a targeted antigen. Based on the promise of the documented phase I trials thus far, other phase I/II Vigil™ (FANG™) trials are currently in progress for patients with melanoma, colorectal carcinoma and various other advanced solid tumors. 

## 7. Conclusions and Future Perspectives

Since the discovery of TGFβ1 more than 30 years ago, significant research efforts have been focused on understanding the biology of this potent regulatory cytokine. During this time, much has been learned about TGFβ1’s role in regulating a diverse array of physiologic processes, both in the steady state and in the development of disease. Its dysregulation in cancer specifically has emerged as a major driver of tumor progression, and TGFβ1 is now known to influence several hallmarks of cancer that include angiogenesis, tissue invasion, metastasis and immune suppression. In particular, our understanding of TGFβ1-mediated immune suppression in cancer has provided significant insights into tumor immune escape and has paved the way for therapeutic strategies that aim to improve the efficacy of immune-based cancer treatment modalities. Many of these strategies have shown promise in preclinical models and even in early clinical trials, particularly as technologies have emerged to modify TGFβ1 activity in specific cell populations. As we continue to learn more about the pleiotropic activities of TGFβ1 and the context-dependent nature of these activities within the tumor microenvironment, and as new advances in genetic engineering and genome editing continue to emerge, novel approaches for both therapeutic delivery and TGFβ1 targeting are likely to improve the quality of anti-tumor immune responses in cancer patients. Data obtained from ongoing/future clinical trials and new preclinical studies will also be important for (1) gaining insights into factors that regulate the efficacy of TGFβ1-targeted therapies and (2) identifying patient populations most likely to benefit from such therapies. Moreover, an improved understanding of factors that limit the efficacy of TGFβ1-targeted regimens in some patients might also suggest combinatorial approaches for therapy that may improve treatment outcome for cancer patients in the future. 

## Figures and Tables

**Table 1:** Ongoing cancer clinical trials targeting TGFβ1 to improve immunotherapy. Clinical trial information obtained from ClinicalTrials.gov.

Trial Identifier	Description of Therapy	Cancer	Status
*Adoptive Cell Transfer Therapies*			
NCT00368082	LMP-specific DNR-CTL	EBV+ lymphoma	Phase I; ongoing, not recruiting
NCT02065362	LMP/BARF1/EBNA1-specific DNR-CTL ± lymphodepletion	EBV+ nasopharyngeal carcinoma	Phase I; currently recruiting
NCT00889954	HER2 CAR/EBV-specific DNR-CTL	Advanced stage HER2+ malignancies	Phase I; ongoing, not recruiting
NCT02379520	E6/E7-specific DNR-CTL	HPV-related/HPV+ cancers	Phase I; recruiting
NCT01955460	Lymphodepletion + DNRII TIL + high-dose IL-2	Melanoma	Phase I; recruiting
*Autologous Tumor Cell Vaccines*			
NCT01061840	Vigil™ (FANG™) bi-shRNAfurin + GM-CSF vaccine	Ewing sarcoma, non-small cell lung cancer, liver cancer, thyroid cancer	Phase I; ongoing, not recruiting
NCT01453361	Vigil™ (FANG™) bi-shRNAfurin + GM-CSF vaccine	Advanced melanoma (Stage IIIc/IV)	Phase II; ongoing, not recruiting
NCT01505166	Vigil™ (FANG™) bi-shRNAfurin + GM-CSF vaccine	Colorectal carcinoma with liver metastases	Phase II; ongoing, not recruiting
